# Sperm Autoantigenic Protein 17 Predicts the Prognosis and the Immunotherapy Response of Cancers: A Pan-Cancer Analysis

**DOI:** 10.3389/fimmu.2022.844736

**Published:** 2022-05-03

**Authors:** Zewei Tu, Jie Peng, Xiaoyan Long, Jingying Li, Lei Wu, Kai Huang, Xingen Zhu

**Affiliations:** ^1^ Department of Neurosurgery, The Second Affiliated Hospital of Nanchang University, Nanchang, China; ^2^ Jiangxi Key Laboratory of Neurological Tumors and Cerebrovascular Diseases, Nanchang, China; ^3^ Institute of Neuroscience, Nanchang University, Nanchang, China; ^4^ Jiangxi Health Commission (JXHC) Key Laboratory of Neurological Medicine, Nanchang, China; ^5^ The Second Clinical Medical College of Nanchang University, Nanchang, China; ^6^ East China Institute of Digital Medical Engineering, Shangrao, China; ^7^ Department of Comprehensive Intensive Care Unit, The Second Affiliated Hospital of Nanchang University, Nanchang, China

**Keywords:** sperm autoantigenic protein 17 (SPA17), pan-cancer, prognostic biomarker, immunotherapy response, CMap

## Abstract

**Background:**

Sperm autoantigen protein 17 (SPA17) is a highly conserved mammalian protein that participates in the acrosome reaction during fertilization and is a recently reported member of the cancer-testicular antigen (CTA) family. It has been reported that the SPA17 expression is limited in adult somatic tissues and re-expressed in tumor tissues. Recently, studies have found that SPA17 regulates the progression of various cancers, but its role in cancer immunotherapy is not clear.

**Methods:**

The pan-cancer and normal tissue transcriptional data were acquired from The Cancer Genome Atlas (TCGA) and the Genotype-Tissue Expression (GTEx) datasets. We explored the SPA17 pan-cancer genomic alteration analysis in the cBioPortal webtool. The Human Protein Atlas (HPA) and ComPPI websites were used to mine the SPA17 protein information. We performed a western blotting assay to validate the upregulated SPA17 expression in clinical glioblastoma (GBM) samples. The univariate Cox regression and Kaplan–Meier method were used to assess the prognostic role of SPA17 in pan-cancer. Gene Set Enrichment Analysis (GSEA) was used to search the associated cancer hallmarks with SPA17 expression in each cancer type. TIMER2.0 was the main platform to investigate the immune cell infiltrations related to SPA17 in pan-cancer. The associations between SPA17 and immunotherapy biomarkers were performed by Spearman correlation analysis. The drug sensitivity information from the Connectivity Map (CMap) dataset was downloaded to perform SAP17-specific inhibitor sensitivity analysis.

**Findings:**

SPA17 was aberrantly expressed in most cancer types and exhibited prognosis predictive ability in various cancers. In addition, our results also show that SPA17 was significantly correlated with immune-activated hallmarks (including pathways and biological processes), immune cell infiltrations, and immunoregulator expressions. The most exciting finding was that SPA17 could significantly predict anti-PDL1 and anti-PD1 therapy responses in cancer patients. Finally, specific inhibitors, like irinotecan and puromycin, which correlate with SPA17 expression in different cancer types, were also screened using Connectivity Map (CMap).

**Conclusions:**

Our results reveal that SPA17 was abnormally expressed in cancer tissues, and this expression pattern could be associated with immune cell infiltrations in tumor microenvironments. Clinically, SPA17 not only acted as a potent prognostic factor to predict the clinical outcomes of cancer patients but was also a promising immunotherapy predictive biomarker for cancer patients treated with immune-checkpoint inhibitors (ICIs).

## Introduction

Cancer-testicular antigen (CTA), a subgroup of proteins usually expressed in male reproductive tissues, is found re-expressed in various cancer tissues along with malignancy progression. Although many studies have revealed that different CTAs regulate the proliferation, migration, and invasion of cancer cells (1), CTAs have also been proven as reliable biomarkers for cancer diagnosis and prognosis prediction. In recent years, the function and importance of CTAs in tumorigenesis have attracted much attention. Because of their limited expression in normal tissues, reexpression in cancer tissues, and immunogenicity, researchers have realized that CTAs could be a possible target for adoptive T cell therapy (ACT) (2). Because most CTAs are intracellular proteins, efforts to explore new cellular immunotherapies based on CTA have focused on isolating and expanding circulating CTA-specific T cells to achieve the engineering of antigen-specific TCR-T cells (3–5).

SPA17 (sperm autoantigen protein 17), originally found in sperm flagellar fibrous sheath (6), is a CTA that encodes a protein located on the cell surface (7). The highly conserved N-terminal sequence of SPA17 and the presence of an A-kinase-anchoring protein (AKAP)-binding motif within this region suggest that SPA17 may function independently of protein kinase A (PKA) in tumor cells separate from the AKAP complex (8). The carbohydrate-binding motif at the core of SPA17 may mediate intercellular adhesion and thus be involved in lymph node migration and invasion of tumor cells (9, 10). As a type of CTA, recent studies have also found SPA17 expression in various tumors, including ovarian cancer, breast cancer, multiple myeloma, cervical cancer, endometrial cancer, and non-small cell lung cancer (11–13). Because the Sp17 protein contains functional cytotoxic T lymphocyte (CTL) epitopes, it has the potential to be a target for tumor vaccines. Chiriva-Internati, Maurizio et al. have shown that SPA17 was a new CTA of multiple myeloma (MM) and a suitable target for its tumor vaccine (14). In addition, the authors’ studies on ovarian cancer (OC) reached similar conclusions (15). Another study on ovarian cancer showed that SPA17 could be used as a new biomarker for OC immunotherapy (16). However, the biological functions and clinical implications of SPA17 in cancers are still unclear and need to be further clarified.

SPA17 is not expressed in normal tissues except in the testis and ciliated cells (17), but it is activated in malignant tumors. The limited expression of normal tissues makes SPA17 ideal for targeting immune infiltrating cells in cancers. Considering the special role of SPA17 in cancers and its potential role in cancer immunity, we designed the pan-cancer analysis to investigate the aberrant expression, prognostic role, and immunotherapy predictive ability of SPA17 in various cancers. This study used transcriptomic data of the TCGA-Pan-cancer cohort and GTEx dataset to reveal the SPA17 expression difference between normal human and tumor tissues, and a western blot was performed to detect the upregulated expression of SPA17 in clinical glioblastoma (GBM) samples. The univariate Cox regression and Kaplan–Meier method were used to determine the prognostic role of SPA17 in each cancer. Gene Set Enrichment Analysis (GSEA) revealed the enriched cancer hallmarks associated with SPA17 expression. Multiple immune cell infiltration quantitative methods, including CIBERSORT, XCELL, MCPCounter, EPIC, and TIDE, were used to quantify the cell infiltration levels of each cancer sample and calculate the correlations between immune cells infiltration and SPA17 expression in each cancer. Finally, two independent cancer cohorts consisting of patients that received immune checkpoint blockade (ICB) therapy were used to examine the predictive role of SPA17 in cancer patient prognosis and immunotherapy response.

## Methods and Materials

### Data Source and Processing

Cancer tissue transcriptional data from the TCGA-Pancancer cohort and normal human tissue data from the Genotype-tissue expression (GTEx) database were downloaded from UCSC Xena (https://xenabrowser.net/) (18). The expression profiles were transferred to transcripts per kilobase million (TPM) format, and the log2(TPM+1) format data were used for subsequent analysis. Twenty-two of the thirty-three cancer types had matched normal tissue data using the matched information from Gene Expression Profiling Interactive Analysis 2 (GEPIA2; http://gepia2.cancer-pku.cn/#dataset) website (19). The abbreviations of cancers are presented in **Supplemental Table 1**.

### Genomic Alterations Analysis of SPA17 in Human Cancers

The cBioPortal (http://www.cbioportal.org) is a multifunctional cancer genomics database that can identify the molecular data in cancer tissues and understand the associated genetics, epigenetics, gene expression, and proteome (20, 21). In this study, we used cBioPortal to investigate the genomic alteration (including mutation, structural variation, amplification, deep deletion, and multiple alterations) rates of SPA17 across cancers. We also visualized the genomic alteration rate using the bar plots through the cBioPortal webtool.

### SPA17 Protein Localization and Interaction

The protein expression profiles of human tissue types are displayed in Human Protein Atlas (HPA; www.proteinatlas.org) (22). We used the immunofluorescence staining images of two human cancer cell lines (A-431 and U251) to show the subcellular localization of SPA17 in cancer cells. ComPPI (https://comppi.linkgroup.hu/) is a novel, integrated and open-source protein–protein interaction (PPI) database that integrates information from multiple databases and provides qualitative information about interactions, proteins, and their localization (23). We downloaded the PPI data of SPA17 from the ComPPI website and annotated the protein information using the “ID mapping” function of the UniProt database (https://www.uniprot.org/).

### GBM Samples Collection and Western Blot Assay

A total of seven GBM samples, including adjacent tissues, were removed from patients hospitalized in the Department of Neurosurgery of the Second Affiliated Hospital of Nanchang University (NCUSAH) in 2021. After the tumor excisions were removed from patients with GBM, they were stored in liquid nitrogen. Informed consent was acquired from the inpatients enrolled in this study. The use of clinical excisions was consented to by the Medical Ethics Committee of NCUSAH. The clinical sample collection and usage were in strict accordance with the guideline. The primary SPA17 and beta-tubulin rabbit polyclonal antibodies were obtained from the Proteintech company (13367-1-AP, 10094-1-AP), and the concentrations of antibodies were entirely following their specifications. The western blot assay was carried out according to the process described in our previous study (24). The agents and related kits used in this research were consistent with the previous study.

### Prognostic Analysis

Related prognostic information, including overall survival (OS) time, progression-free survival (PFS) time, disease-free survival (DFS) time, and disease-specific survival (DSS) time, were also downloaded from the UCSC Xena database. Then Kaplan–Meier model and univariate Cox regression were used to assess the prognostic role of SPA17 for a specific prognosis type in each cancer. The SPA17 continuous variable expression data were used in the univariate Cox regression, and bivariate SPA17 expression levels were used to perform Kaplan–Meier curves analysis with the cut-off chosen by the “surv-cutpoint” function of the “survminer” R package (0.4.9). Finally, the log-rank *P*-value of the K-M method and hazard ratio (HR) with a 95% confidence interval (95% CI) were computed, and the outcomes were summarized and presented in a heatmap.

### Identification of Differentially Expressed Genes (DEGs) Between Low- and High-SPA17 Subgroup

To determine the DEGs between low- and high-SPA17 subgroups for each cancer, the cancer patients were ranked by SPA17 mRNA expression, with the top 30% of patients defined as the high-SPA17 subgroup and the bottom 30% as the low-SPA17 subgroup. Differential expression analyses were performed between low- and high-SPA17 subgroups using the “limma” R package (25), the log2 (fold change), and the adjusted *P*-value of each gene for each cancer type. The genes with *P*-adjusted values < 0.05 were regarded as DEGs. The DEGs between the low- and high-SPA17 subgroup for each cancer are shown in **Supplementary Table 2**.

### Gene Set Enrichment Analyses

The “gmt” file of the hallmark gene set (h.all.v7.4.symbols.gmt) containing 50 hallmark gene sets was downloaded from the website of Molecular Signatures Database (MSigDB, https://www.gsea-msigdb.org/gsea/index.jsp) and used to calculate the Normalized Enrichment Score (NES) and False Discovery Rate (FDR) of each biological process for each cancer type. The Gene Set Enrichment Analysis was conducted using the R packages “clusterProfiler,” and “GSVA” (26, 27), and the results are summarized in the bubble plot depicted by the R package “ggplot2.”

### Immune Cell Infiltration Analysis of SPA17

Tumor IMmune Estimation Resource (TIMER) is a data source for analyzing immune cell infiltrations across distinct cancers using various methods (28). The SPA17-associated immune cell infiltration correlations of the TCGA Pan-cancer project were downloaded from the TIMER2.0 database (http://timer.cistrome.org/) in the “Gene” function of the “Immune Association” section. We visualized the statistical Spearman correlations between SPA17 mRNA expression and 21 immune cell subsets, including cancer-associated fibroblast (CAF), progenitors of lymphoid, B cells, neutrophils, hematopoietic stem cells (HSC), CD4+ T cells, progenitors of myeloid, progenitors of monocyte, endothelial cells (Endo), eosinophil (Eos), regulatory T cells (Tregs), T cell follicular helper, NK T cells, γ/δ T cells, monocytes, macrophages, dendritic cells, CD8+ T cells, mast cells, and NK cells across cancers in a heatmap by using the R package “ggplot2”.

### Immunotherapy Prediction Analysis

The Spearman correlation analysis was performed to show the associations between SPA17 and reported biomarkers of cancer immunotherapy for each cancer type. The relationship between SPA17 and tumor mutational burden (TMB) and microsatellite instability (MSI) was also analyzed in pan-cancer by Spearman correlation analysis. To explore the relationship between SPA17 and immune checkpoint blockade (ICB) therapy response, two immune checkpoint blockade (ICB) therapy cohorts were obtained to validate the immunotherapy response prediction ability of SPA17. The IMvigor210 cohort contains 298 urological cancer patients treated with atezolizumab (anti-PDL1), and the GSE91061 cohort includes 26 melanoma patients’ transcriptomic profiles before receiving nivolumab (anti-PD1). Patients were divided into a SPA17 low-expression group and a high-expression group according to the best cut-off value using the “surv-cutpoint” function of the “survminer” R package (0.4.9). A Chi-square test was used to evaluate the proportion difference of responders between low- and high-SPA17 cancer groups.

### Connectivity Map Specific Inhibitor Analysis

The Connectivity Map (CMap), an essential database in pharmacogenomics, can be used to connect diseases with the drugs that treat them (29). CMap (https://portals.broadinstitute.org/cmap/) was used to identify the relationships between SPA17 and specific inhibitors that correlated with SPA17 expression for each cancer type. The methods and processes of applying the web tool and visualizing the heatmap were completed according to the previous study (30). The statistically significant components correlated with SPA17 expression in each cancer type are summarized in **Supplementary Table 3**.

### Statistical Analyses

The Wilcoxon rank-sum test was performed to calculate statistical significance and compare the SPA17 expression levels between tumor and normal tissues. A Paired t-test was used to evaluate the statistical significance between the SPA17 protein expression levels in clinical GBM samples and adjacent tissues. Univariate Cox regression analysis and the Kaplan–Meier method (log-rank test) were employed to assess the prognostic role of SPA17 expression in each cancer. Spearman correlation analysis was performed to evaluate the statistical relationships between SPA17 and other factors, such as immune cell infiltration levels, immune regulator genes, TMB, and MSI. Finally, the chi-square test was used to compute the statistical significance to compare the proportions of ICI-therapy responders and non-responders between the low- and high-SPA17 cancer subgroups.

## Results

### SPA17 Is Aberrantly Expressed in Cancer Tissues

**Figure 1:**
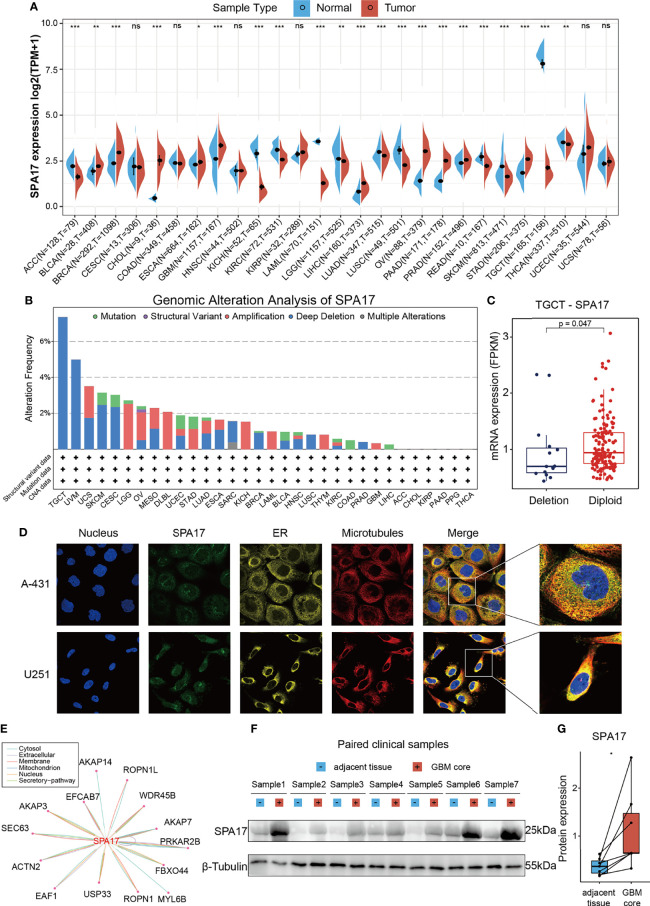
Basic information of SPA17. **(A)** The level of SPA17 expression between tumor and normal tissues in each type of cancer based on the integrated data from TCGA and GTEx datasets. **(B)** Analysis of SPA17 change frequency in pan-cancer research according to cBioPortal database. **(C)** The SPA17 expression levels between SPA17-deletion and diploid TGCT patients. **(D)** The immunofluorescence images of SPA17 protein, nucleus, endoplasmic reticulum (ER), microtubules and the incorporative images in A-431 and U251 cell lines. **(E)** The protein-protein interaction (PPI) network presents the proteins interacting with SPA17. **(F, G)** Western blot protein detection of the SPA17 expression levels in paired GBM and adjacent normal tissues. The labelled asterisk indicated the statistical p-value (ns, p > 0.05, *p < 0.05, **p < 0.01 and ***p < 0.001).

To uncover the expression levels of SPA17 across cancers, we integrated the TCGA and GTEx databases to reflect the level of SPA17 mRNA expression in pan-cancer. The results showed a high expression of SPA17 in 10 kinds of tumors: BLCA, BRCA, CHOL, ESCA, GBM, LIHC, OV, PAAD, PRAD, and STAD. In comparison, a low SPA17 expression was noticed in 11 tumors: ACC, KICH, LUAD, KIRC, LAML, LUSC, LGG, READ, TGCT, SKCM, and THCA (**Figure 1A**). Notably, in TGCT, the SPA17 expression was significantly inhibited. This may be related to its ligand Ropporin, a spermatogenic cell-specific protein, which has also been confirmed in previous studies (31, 32). The results indicated that SPA17 expression was unbalanced in cancers, consistent with previous studies.

### Genetic Alteration Proportion of SPA17 Is Lower Than 5% in Most Cancers

Given the abnormal SPA17 expressions observed in the cancers, we speculated if genetic alterations of SPA17 resulted in this phenomenon. Therefore, we conducted a genetic alteration analysis of SPA17 in the TCGA Pancancer tumor samples. As shown in **Figure 1B**, the highest SPA17 alteration frequency (>6%) appeared for patients with testicular germ cell tumors (TGCT) with “Deep Deletion” as the primary type. Therefore, we further analyzed the SPA17 expression in the deletion and the diploid groups of TGCT tumors and found that the expression in the diploid group was significantly higher than that in the deletion group (**Figure 1C**). In contrast, the “Amplification” type of copy number alteration (CNA) was the altered primary type in the brain lower grade glioma cases, which had an alteration frequency of ~2.5% (**Figure 1B**). Notably, all DLBC cases with genetic alteration (~2% frequency) had SPA17 amplification **(** **Figure 1B** **)**. However, in general, the frequency of the genetic alteration of SPA17 was not high, which may be related to the high conservation characteristics of SPA17 (33).

### SPA17 Protein Localization, Interaction, and Expression

To comprehensively understand the SPA17 protein, we obtained the immunofluorescence of SPA17 protein images from the Human Protein Atlas (HPA), downloaded protein-protein interaction information from the ComPPI database, and validated the SPA17 protein expression in clinical GBM samples. The immunofluorescence (IF) images of the cancer cells showed that SPA17 protein was mainly localized in the vesicles of the A-431 and U251 tumor cell lines, according to the HPA database (**Figure 1D**). Next, the PPI network was implemented based on the interaction data obtained from the ComPPI website. It showed the subcellular localization of proteins closely interacting with SPA17 was distributed in the cytosol, mitochondria, nucleus, extracellular, secretory pathway, and membrane (**Figure 1E**). To further validate the expression of SPA17 at the protein level, we used clinical GBM samples. We performed a western blot to confirm the expression of SPA17 protein in GBM samples and adjacent tissues (**Figures 1F, G** **)**. The results showed that SPA17 protein was upregulated in the GBM samples. These data indicate that SPA17 protein is aberrantly expressed and has a potential protein function in cancers.

### Clinical Prognostic Significance of SPA17 in Pan-Cancer

**Figure 2:**
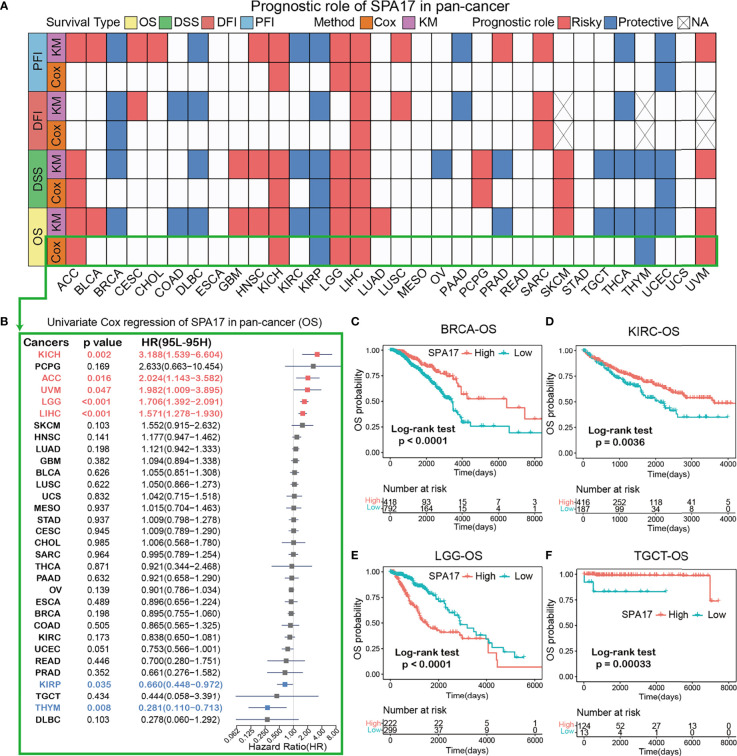
**(A)** Based on the univariate Cox regression and Kaplan-Meier models, summary of the correlation between SPA17 expression and overall survival (OS), disease-specific survival (DSS), disease-free interval (DFI) and progression-free interval (PFI). Red indicates that SPA17 is a risk factor for the prognosis of cancer patients, while blue represents a protective factor. Only p-values < 0.05 are shown. **(B)** The forest plot showed the prognostic role of SPA17 in cancers by univariate Cox regression. The red type of cancer represents SPA17 as a statistically significant risk factor, and blue represents a protective factor. **(C–F)** Kaplan-Meier overall survival curves of SPA17 in BRCA **(C)**, KIRC **(D)**, LGG **(E)** and TGCT **(F)**.

To further investigate the prognostic potential of SPA17 in cancers, four prognostic indicators of 33 cancers were analyzed by the Kaplan–Meier method (log-rank test) and univariate Cox regression. The heatmap is used to show the relationship between SPA17 and prognosis. The results showed that SPA17 was highly correlated with the prognosis of most cancers except ESCA, MESO, READ, STAD, and UCS (**Figure 2A**). Specifically, SPA17 was a risk factor for poor prognosis of ACC, BLCA, CHOL, CESC, HNSC, GBM, KICH, LGG, LIHC, LUSC, LUAD, PCPG, SARC, SKCM, and UVM. In addition, SPA17 was associated with a good prognosis of BRCA, COAD, DLBC, KIRC, KIRP, PAAD, OV, PRAD, THCA, TGCT, THYM, and UCEC. It is worth noting that SPA17 was a risk factor for four prognostic survival indicators of LIHC, which was significantly correlated with poor prognosis. At the same time, we found that SPA17 was a protective factor for four prognostic types of BRCA, DLBC, KIRP, and THCA tumors using a log-rank test statistical method.

The results shown in the forest plot (**Figure 2B**) demonstrate the downregulation of SPA17 expression was related to the time delay of OS: KICH (HR = 3.188[95% CI, 1.539–6.604], *P* = 0.002), ACC (HR = 2.024 [95% CI, 1.143–3.582], *P* = 0.016), UVM (HR = 1.982 [95% CI, 1.009–3.895], *P* = 0.047), LGG (HR = 1.706 [95% CI, 1.392–2.091], *P* < 0.001), LIHC (HR = 1.571 [95% CI, 1.278–1.930], *P* < 0.001). The upregulation of SPA17 expression was related to the time delay of OS: KIRP (HR=0.660 [95% CI, 0.448–0.972], *P*=0.035) and THYM (HR = 0.281 [95% CI, 0.110–0.713], *P*=0.008).

Many studies have suggested that SPA17 is closely related to the prognosis and progression of BRCA and TGCT (10, 32). Therefore, we analyzed the Kaplan–Meier curve of BRCA and TGCT and found that a higher expression of SPA17 was related to a better survival outcome (**Figures 2C, F** **)**, indicating that SPA17 was a prognostic biomarker of OS in BRCA and TGCT. In addition, we also found that the high expression of KIRC had a good prognosis (**Figure 2D**). Additional Kaplan–Meier survival analysis also confirmed that a higher SPA17 expression in LGG was related to a poor OS prognosis (**Figure 2E**). SPA17 had a prognostic role in predicting the prognosis of cancers, but the roles were complicated and multifaceted across cancers. Further investigation should focus on the function of the SPA17 protein in cancer cells.

### Gene Set Enrichment Analysis of SPA17 Reveals Its Association With the Cancer Immune Response

To excavate the biological processes associated with SPA17 expression in cancers, we performed differential expression analysis between the top 30% SPA17 expression subgroup and bottom SPA17 expression subgroup in each cancer type. The differential expression genes (DEGs) in each cancer type are summarized in **Supplemental Table 2**.

**Figure 3:**
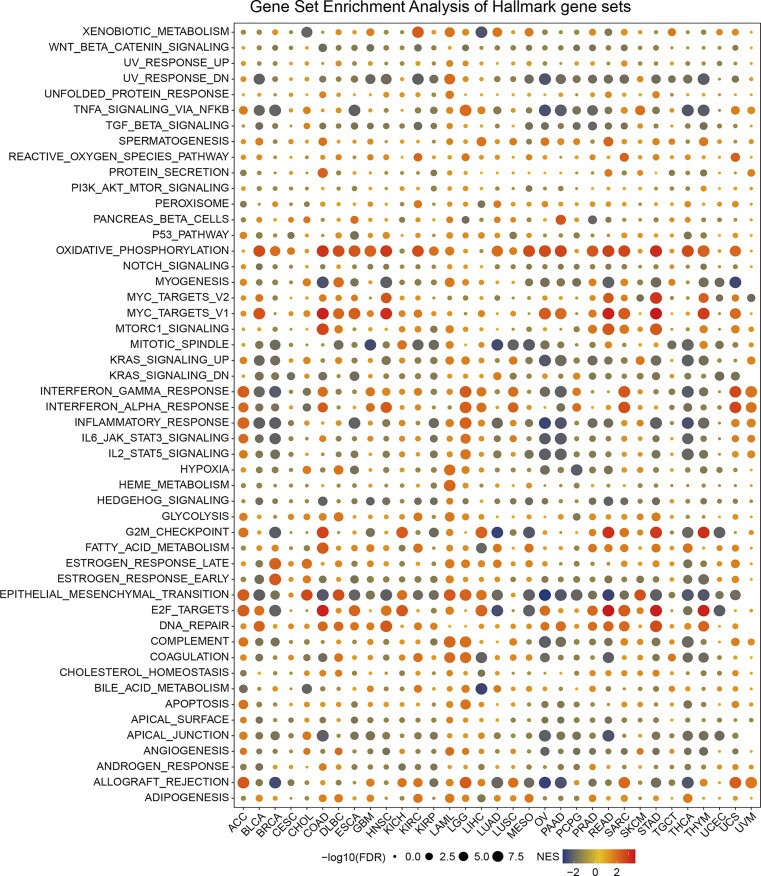
The hallmarks gene set enrichment analysis (GSEA) of SPA17 in pan-cancer. The size of the circle represents the false discovery rate (FDR) value of each cancer enrichment item, and the color represents the normalized enrichment score (NES) of each enrichment item.

Based on the DEGs between the high and low-SPA17 subgroups, we performed GSEA analysis across 33 cancer types to evaluate the SPA17-associated cancer hallmarks. We found that immune-related pathways: TNFA-signaling-via-NFKB, IFN-α response, IFN-γ response, allograft-rejection pathways, and inflammatory response were remarkably enriched in a variety of tumors, especially in ACC, BLCA, BRCA, LGG, UCS, and UVM. These results indicate that SPA17 could be closely related to the tumor immune microenvironment and ligand–receptor interactions between malignant tumor cells and immune cells. Moreover, we also found that SPA17 was associated with the epithelial-mesenchymal transformation of many kinds of tumors and had a significant positive correlation with ACC, CHOL, DLBC, KICH, LAML, LGG, LIHC, and SKCM, suggesting that SPA17 could play an important role in tumor invasion and migration. Furthermore, oxidative phosphorylation, MYC targets, E2F targets, and androgen response were also closely related to the expression of SPA17 in cancers (**Figure 3**). In summary, these results provide evidence that the abnormal expression of SPA17 may be involved in the immune response of cancers. SPA17 was deeply involved in the occurrence and development of cancer, which led us to study further the role of SPA17 in the cancer-immune response and microenvironment.

### Immune Cell Infiltration Analyses of SPA17 Across Cancers

**Figure 4:**
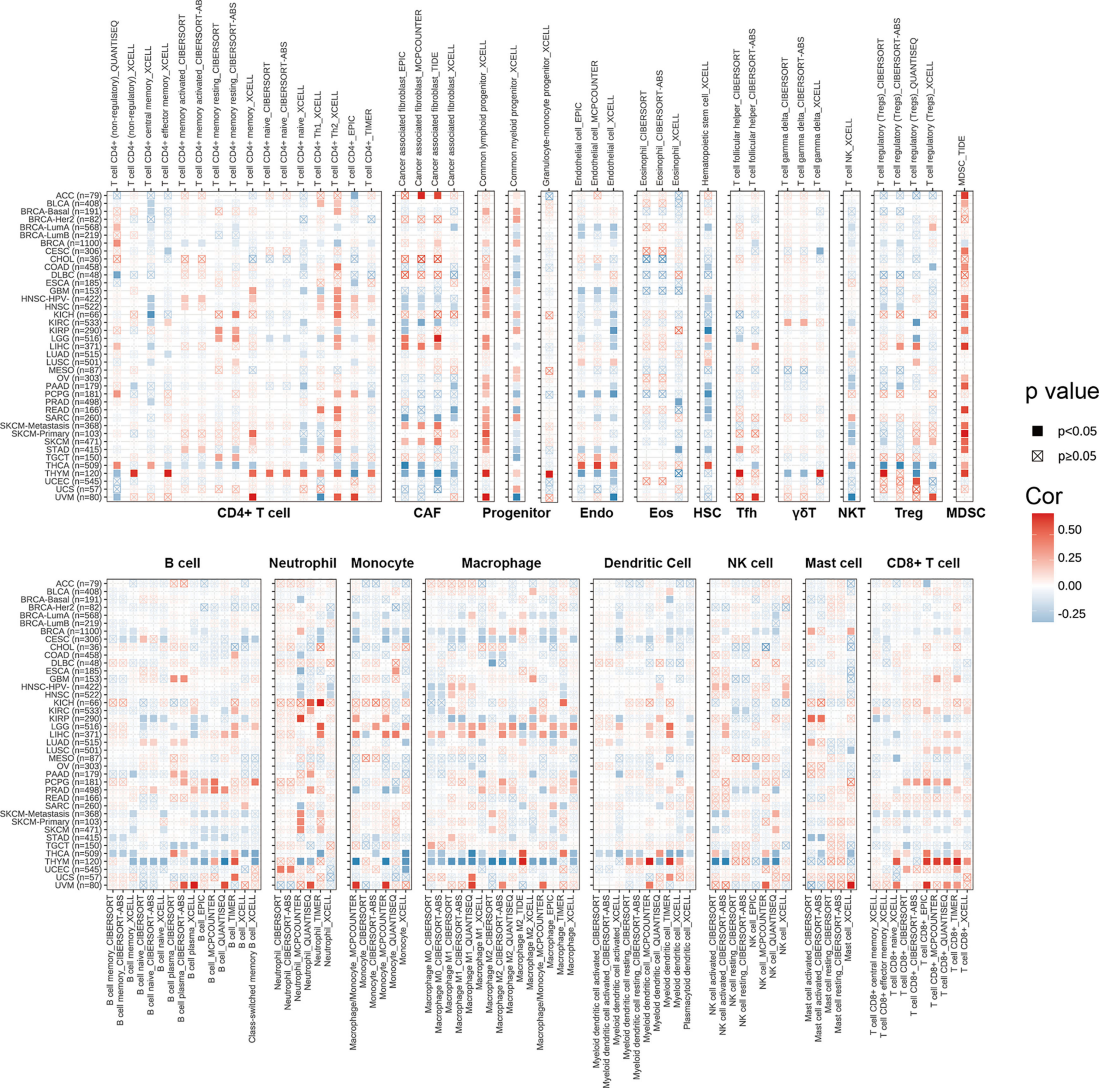
The correlations of SPA17 expression and the infiltration levels of CD4+ T cells, CAF, progenitor, Endo, Eos, HSC, Tfh, γδT, NKT, regulatory T cells (Tregs), B cells, neutrophils, monocytes, macrophages, dendritic cells, NK cells, Mast cells and CD8+ T cells in cancers. Positive correlation in red and negative correlation in blue.

We explored the correlations between SPA17 expression and immune cell infiltration levels across cancers according to the above conclusion. We employed the TIMER2.0 database to show the landscape of SPA17 associated with various immune cell infiltrations, which were conducted in a variety of quantitative immune infiltration platforms in cancers. The results reveal infiltration levels of CD4+ T cells, cancer-associated fibroblast (CAF), progenitors of lymphoid, progenitors of myeloid, endothelial cell (Endo), eosinophil (Eos), hematopoietic stem cell (HSC), T cell follicular helper (Tfh), T cell gamma delta (γ/δT), nature killer T cell (NKT), T cell regulatory (Tregs), myeloid-derived suppressor cell (MDSC), neutrophils, monocytes, B cells, dendritic cells, macrophages, mast cells, NK cells, and CD8+ T cells in pan-cancer (**Figure 4**). In general, SPA17 was positively correlated with the level of immune infiltration of many kinds of infiltrating cells, such as MDSC, progenitors of lymphoid, CD8+ T cell, and CD4+ T cell in various cancers. Likewise, we found SPA17 was significantly correlated with multiple infiltrating immune cells, such as macrophages, B cells, CAF, and CD8+ T cells, in thymic carcinoma (THYM) and thyroid carcinoma (THCA). However, the trend of this correlation was slightly different, which may be due to the other immune infiltration rates of certain tumors.

### Correlations Between SPA17 and Immunomodulators, TMB, and MSI

**Figure 5:**
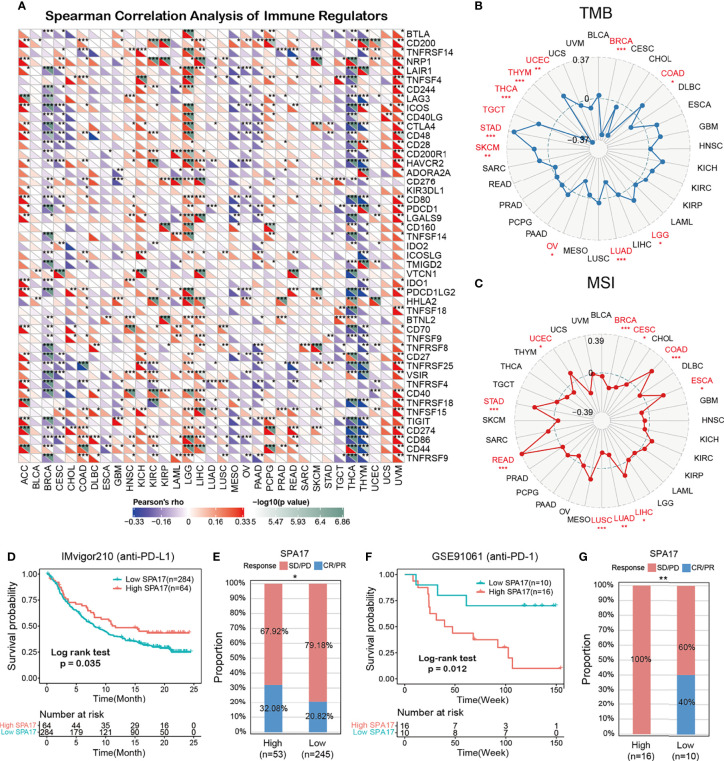
**(A)** The Spearman correlation heatmap shows the correlation between the expression of SPA17 and 47 kinds of immune regulators in pan-cancer. Red represents positive correlation and blue represents negative correlation. **(B)** Correlations between SPA17 expression and tumor mutation burden in pan-cancer. **(C)** Correlations between SPA17 expression and microsatellite instability in pan-cancer. **(D)** Kaplan-Meier curve of low and high-SPA17 subgroup in IMvigor210 cohort (anti-PD-L1), and the proportion of tumors (including kidney cancer) in the low-SPA17 and high-SPA17 subgroups in the IMvigor210 cohort who responded to PD-1 therapy. **(E)** Kaplan-Meier curve of GSE91061 (anti-PD-L1 melanoma) in the low and high-SPA17 patient groups, and the proportion of melanoma patients in the GSE91061 low and high-SPA17 subgroups that responded to anti-PD-1 therapy. The labelled asterisk indicated the statistical p-value (*p < 0.05, **p < 0.01, ***p < 0.001).

We also performed Spearman correlation analysis to uncover the associations between SPA17 expression and 47 immunomodulatory genes (including immune checkpoint genes and immune cell marker genes) in pan-cancer, as shown in **Figure 5A**. We found that SPA17 was positively correlated with most of the immunomodulatory factors of ACC, LGG, and UVM but negatively correlated with most of BRCA, CESC, PAAD, THYM, and THCA. To understand the role of SPA17 in predicting the efficacy of immune checkpoint inhibitor (ICI) therapy, we assessed the correlation between the expression of SPA17 and two well-known immunotherapy predictive biomarkers (34, 35), tumor mutation burden (TMB) and microsatellite instability (MIS). The results showed that the SPA17 expressions of COAD, LGG, SKCM, STAD, TGCT, and UCEC were positively correlated with the TMB value and were negatively correlated with the TMB of BRCA, LUAD, and THCA (**Figure 5B**). In addition, a positive correlation was found between the expressions of SPA17 and MSI in COAD, ESCA, LIHC, READ, STAD, and UCEC; a negative correlation was found in BRCA, CESC, LUAD, and LUSC **(** **Figure 5C** **)**. Our results suggest that SPA17 could have the power to predict the efficacy of ICIs in corresponding cancers.

### SPA17 Predicts the Response to Cancer Immunotherapy

ICIs, such as anti-PD-L1, anti-PD-1, and anti-CTLA-4 antibodies, have contributed to immunotherapy in cancers (36). Therefore, according to the clues mentioned above, we analyzed the predictive role of SPA17 in patients with cancer who received ICI therapy. The relationship between SPA17 and the response to anti-PDL1 treatment in patients with urinary tumors showed that the survival rate and time of patients with high SPA17 expression were more favorable than those with low SPA17 expression (**Figure 5D**). In the urinary system tumors of the IMvigor210 cohort, the anti-PD-L1 response rate of patients with high SPA17 expression was 32.08%, which was significantly higher than 20.82% of patients with low SPA17 expression. In addition, the opposite results were found in patients with melanoma who received anti-PD-1 therapy. In the GSE91061 melanoma cohort (**Figure 5E**), melanoma patients with low SPA17 expression had a better chance of survival than patients with high SPA17 expression. Furthermore, in the subgroup of patients with high SPA17 expression, the response rate to PD-1 was 0%, while in the subgroup with low SPA17 expression, 40% of patients responded to PD1 therapy. These data confirm the potential ability of SPA17 in the prediction of immunotherapy response and indicate that SPA17 was a promising biomarker for cancer immunotherapy. However, because of our detailed results, further investigations of the predictive role of SPA17 in cancer immunotherapy need to be performed clinically and mechanically in individual cancer types.

### Connectivity Map (CMap) Analysis Reveals SPA17-Associated Drugs

**Figure 6:**
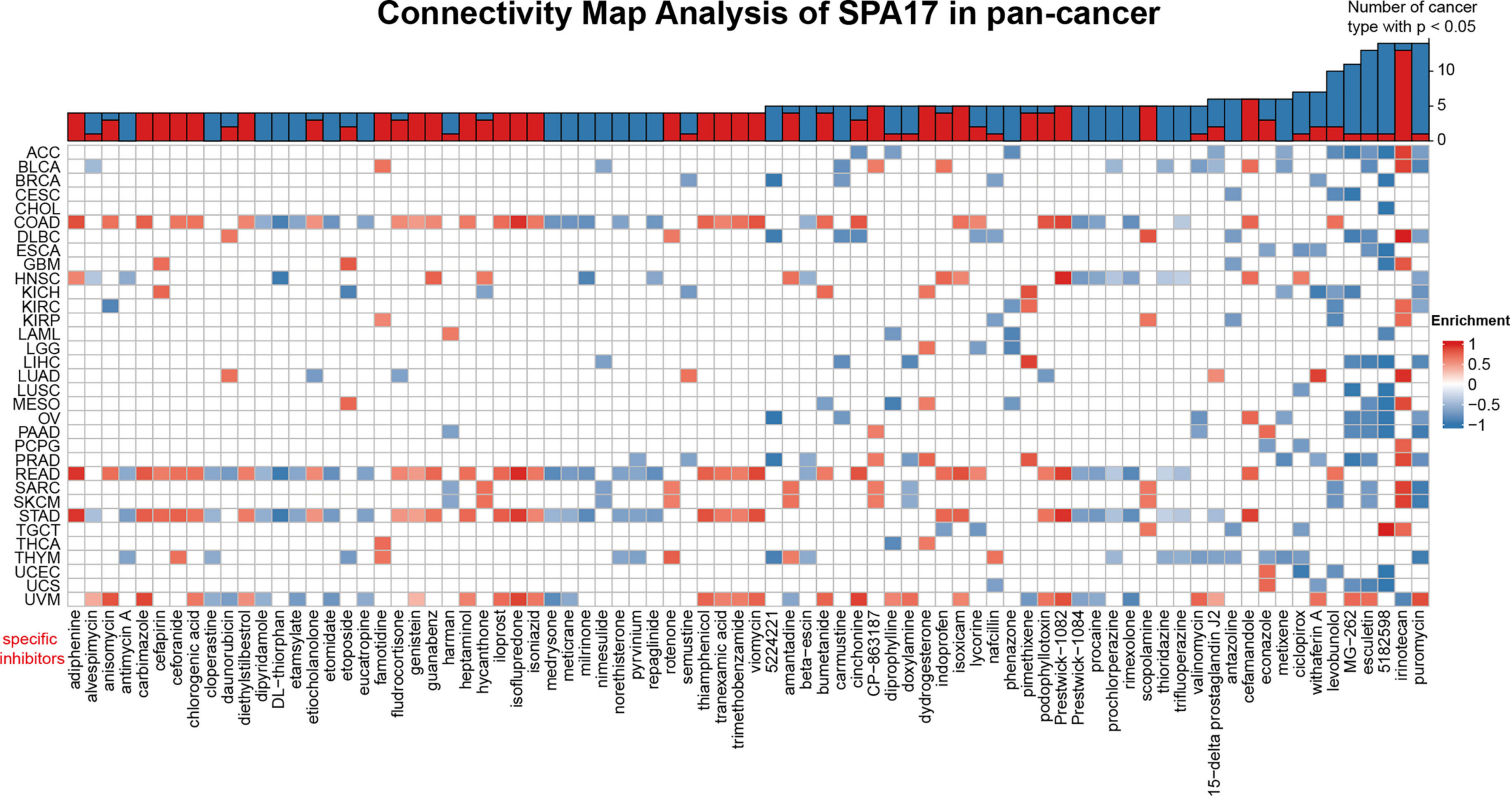
Heatmap representing the rich fraction (positive in blue, negative in red) of each drug in the CMap database of each cancer. Components or drugs are ranked in descending number of enriched cancers from right to left.

To unveil the SPA17-associated inhibitors or components in various cancers, we screened the SPA17-associated drugs using data downloaded from the CMap dataset; the potential elements of SPA17 in pan-cancer are presented in the form of a heatmap in **Figure 6**. The heatmap shows the drugs or ingredients that target SPA17 in more than three cancers, and the detailed parameters of the enriched components in each cancer are listed in **Supplemental Table 3**. Our results suggest that irinotecan was significantly enriched in 14 kinds of cancers and positively correlated with SPA17 in ACC, BLCA, DLBC, GBM, KIRC, KIRP, LUAD, MESO, PCPG, PRAD, SARC, SKCM, TGCT, and especially in DLBC, but only negatively correlated in UVM. Interestingly, puromycin and alkaloid 5182598 were also enriched in 14 cancers. However, in contrast to irinotecan, these two drugs were negatively correlated with 13 cancers, in which puromycin was positively correlated only with UVM, and alkaloid 5182598 was positively correlated with TGCT. Esculetin was significantly enriched in 13 species, and MG-262 was significantly enriched in 12. Both of them were positively correlated with only one kind of tumor. Interestingly, most drugs were significantly enriched in UVM, STAD, and COAD cancers in terms of cancer types, with the enrichment relationships showing a high degree of consistency. These drugs have been used to prevent and treat several cancers in recent years, and the correlations between these components and SPA17 should be examined in further investigations. Our results show that the current clinical application of these drugs was only the beginning, and their role in the development of different cancers, as potential targets, and their potential mechanisms need to be further explored. The underlying mechanisms associated with SPA17 are also worthy of examination.

## Discussion

During the antitumor immune response, the drug’s efficacy largely depends on the functional status of tumor-specific effector immune cells (37–40). The proliferation and activity of effector cells are regulated by immune checkpoint costimulation and co-inhibition signals (41–43). Therefore, the antitumor immune response is often enhanced by promoting the costimulatory signal as positive or blocking the inhibitory signal of negative regulation (44). At present, it has been found that many immune checkpoint molecules can be used for drug therapy, such as programmed cell death 1 ligand 1 (PD-L1), programmed cell death protein 1 (PD-1), cytotoxic T-lymphocyte associated protein 4 (CTLA4), T cell immunoreceptor with Ig and ITIM domains (TIGIT), hepatitis A virus cellular receptor 2 (HAVCR2, also known as TIM-3), and lymphocyte activating 3 (LAG-3) (45–49). CTLA4 and PD-L1/PD-1 were discovered first and studied the most; the combined strategy of anti-PD-1/PD-L1 and anti-CTLA-4 is used in the immunotherapy of many kinds of tumors. However, the curative effect of this combination is not significant in many tumor patients, and it also has strong patient side effects. Therefore, there is an urgent need to study new immune checkpoints (50, 51) and how to predict the cancer immunotherapy effect. In this study, we found SPA17 was a promising biomarker of pan-cancer prognosis, which effectively predicted the response to cancer immunotherapy. Our results provide vital clues for future research on the potential role of SPA17 in tumor immunity and immunotherapy.

First, the expression levels of SPA17 in pan-cancer were analyzed based on the GTEx and TCGA databases. The results suggest that SPA17 was abnormally upregulated in ten cancer types and downregulated in eleven cancer types (**Figure 1A**). However, SPA17 was not associated with genomic alterations in cancers because we found the proportion lower than 5% (**Figure 1B**). Compared with normal testicular tissue, the SPA17 downregulation in TGCT was the most obvious. According to our analysis, the deep deletion of SPA17 resulted in a lower SPA17 expression in patients with TGCT, although few genomic alterations were found in other cancer types (**Figure 1C**). Thus, why SPA17 is unevenly expressed in cancers is unsolved. A study of varicocele sperm shows that a low SPA17 expression of SPA17 was related to mitochondrial damage (52), which is also crucial for the intracellular homeostasis of cancer cells and the cancer therapy effect (53). Mitochondria in cancer cells are characterized by reactive oxygen species (ROS) overproduction, promoting cancer development by inducing genomic instability, modifying gene expression, and participating in signaling pathways (53). This is consistent with the gene set enrichment analysis (GSEA) results showing positive correlations between SPA17 expression and the ROS pathway in cancers. This suggests we further explore why SPA17 was abnormally expressed in cancers and its role in the ROS pathway.

Next, we evaluated the relationship between SPA17 and the clinical prognosis of cancer patients. The OS, DFI, DSS, and PFI analysis results were highly consistent in cancer patients, revealing that SPA17 was significantly related to the prognosis of cancer patients (**Figure 2A**). Specifically, SPA17 was a risk factor for 15 types of cancer patients and a protective factor for 11 cancers. These results show that SPA17 plays an essential role in predicting the prognosis of cancer patients and is expected to become a powerful biomarker of prognosis for cancer patients. Thus, some of our results are consistent with the conclusion of a previous study (32), making our results more reliable.

In our analysis, the GSEA results suggest SPA17 was closely related to immune response associated processes, such as TNFA-signaling *via* NFKB, the IFN-α response, the IFN-γ response, and the inflammatory response (**Figure 3**). Previous studies also underline the potential cancer immunogenicity and immunotherapy targeting the role of the SPA17 protein or transcript in various cancers, such as ovarian cancer, esophageal squamous cell carcinoma, and multiple myeloma (54–56). This information prompts us to consider the role of SPA17 in cancer immunology. Most of these processes were significantly enriched in the cancer subgroups with high SPA17 expression; however, this was not true for BLCA, BRCA, OV, PAAD, THCA, and THYM. These results suggest that SPA17 could play different roles in different types of cancer. Furthermore, recent studies have shown that the inflammatory response, TNFA-signaling *via* NFKB, the IFN-α response, and the IFN- γ response were closely related to the immunotherapy and prognosis of cancer, providing a direction for further exploration of the role of SPA17 in tumors (57–60). Another important finding of our study was the SPA17 expression was highly correlated to the immune infiltration of cancers (**Figure 4**). In most tumors, SPA17was positively associated with the infiltration of MDSC, CD4+ T cells, progenitors of lymphoid, and CD8+ T cells, suggesting that SPA17 was likely to affect tumor development and prognosis by impacting the tumor microenvironment. In addition, the correlation analysis between SPA17 and the pan-cancer immunomodulatory factors suggests that the SPA17 expression was highly correlated to the expression of specific immunomodulatory genes (**Figure 5A**), especially in ACC, BRCA, LGG, THCA, THYM, and UVM. These results indicate SPA17 was most likely involved in cancer progression and prognosis by interacting with cancer microenvironments.

We next explored the relationship between SPA17 expression levels and the response to anti-PD-L1 immunotherapy in a pan-cancer cohort, including kidney renal clear cell carcinoma and the expression levels of anti-PD1 in melanoma (**Figures 5D–G**). The results show that a higher SPA17 expression indicated a better prognosis and more sensitivity to anti-PD-L1 immunotherapy in the IMvigor210 cancer cohort. The opposite was found in patients with melanoma treated with anti-PD-1 (GSE91061 cohort). The cohort with higher SPA17 expression represented a worse prognosis and resistance to anti-PD-1 therapy. However, the treatment responses of both were in line with their survival curves. Our results suggest that SPA17 was an effective biomarker for predicting the response to blocking treatment at immune checkpoints in various cancers. Therefore, we hypothesized that SPA17 could be a powerful and promising biomarker in predicting tumor immunotherapy effects.

Finally, we identified specific inhibitors to screen molecular targets that may eventually lead to new anticancer inhibitors (**Figure 6**). Previous studies have shown that puromycin is an effective translation inhibitor that binds to ribosomes in all fields of life (61). Alkaloid 5182598 is an important anticancer drug (62) that repairs the damaged metabolic pathway in metastatic prostate cancer and, therefore, is a potential therapeutic drug for treating metastatic prostate cancer (63). In the past 25 years, the cytotoxic drug irinotecan has been widely used to treat metastatic or advanced solid tumors (64). However, the underlying mechanism of how puromycin, irinotecan, alkaloid 5182598, and other SPA17-related drugs affect the pan-cancer tumor microenvironments remains to be found. The role of SPA17 in the anticancer activities of these components should also be investigated.

Our research has several limitations that should be considered. Although we identified the potential drugs associated with SPA17 by CMAP analysis, proof of direct interaction between SPA17 and these components and the potential mechanisms is unclear. Furthermore, most of our pan-cancer research data come from online open databases, potentially producing systematic bias with a lack of large-scale clinical cohort data to verify it. At the same time, the SPA17 mechanism affecting the occurrence and development of tumors needs to be further clarified based on experiments. Moreover, the potential drugs to be combined with SPA17 and analyzed by CMAP need to be explained and verified by further mechanism studies.

In conclusion, we conducted a comprehensive SPA17 pan-cancer analysis, showing its potential function as a cancer prognosis biomarker and effective immunotherapy response prediction. The abnormal expression of SPA17 was related to the prognosis, immune regulation, immune cell infiltration, tumor microenvironment, TMB, and MSI of many kinds of tumors. Recently, CTA-targeted antibodies, vaccines, and chimeric antigen receptor-modified T cells (CAR-T) have been used in cancer therapy, with promising results in preclinical and early clinical trials (65). Our study demonstrates that SPA17 can serve as an important prognostic marker in clinical practice. Immunological studies also showed that, as a powerful CTA, SPA17 may be a novel target for various tumor immunotherapies in the future (66). We concluded that SPA17 predicts the prognosis and immunotherapy effects of cancers and is a potential immunotherapy target.

## Data Availability Statement

The original data used in this project can be downloaded in the UCSC (https://xenabrowser.net/datapages/) website.

## Ethics Statement

The studies involving human participants were reviewed and approved by The Ethics Committee of the Second Affiliated Hospital of Nanchang University. The patients/participants provided their written informed consent to participate in this study. Written informed consent was obtained from the individual(s) for the publication of any potentially identifiable images or data included in this article.

## Author Contributions

KH, XZ, and LW designed this study and revised the manuscript; ZT, JP, and JL conducted the data collection, bioinformatic and statistical analysis, figures visualization and manuscript writing. XL revised the manuscript. All of coauthors have approved the final version of manuscript.

## Funding

The research project is supported by the National Natural Science Foundation (grant nos. 81860448, 82002660 and 82172989), the Natural Science Foundation of Jiangxi Province (grant no.20192BAB205077 and 20202ACB216004), the Jiangxi Key research and development projects-Key Project (20212BBG71012), Jiangxi Provincial Science and Technology Innovation Base Plan-Provincial Key Laboratory (20212BCD42008), Construction of Science and Technology Innovation Base-Clinical Medicine Research Center (2021ZDG02001), Jiangxi Key research and development projects (20212BBG73021), Province-Youth Talent Project (20212BCJ23023).

## Conflict of Interest

The authors declare that the research was conducted in the absence of any commercial or financial relationships that could be construed as a potential conflict of interest.

## Publisher’s Note

All claims expressed in this article are solely those of the authors and do not necessarily represent those of their affiliated organizations, or those of the publisher, the editors and the reviewers. Any product that may be evaluated in this article, or claim that may be made by its manufacturer, is not guaranteed or endorsed by the publisher.
